# CUMULATIVE LONELINESS AND ALL-CAUSE MORTALITY AMONG MIDDLE-AGED AND OLDER ADULTS IN THE UNITED STATES, 1996–2019

**DOI:** 10.1093/geroni/igad104.0670

**Published:** 2023-12-21

**Authors:** Xuexin Yu, Tsai-Chin Cho, Ashly Westrick, Chen Chen, Kenneth Langa, Lindsay Kobayashi

**Affiliations:** University of Michigan School of Public Health, Ann Arbor, Michigan, United States; University of Michigan School of Public Health, Ann Arbor, Michigan, United States; University of Michigan School of Public Health, Ann Arbor, Michigan, United States; University of Michigan School of Public Health, Ann Arbor, Michigan, United States; University of Michigan, Ann Arbor, Michigan, United States; University of Michigan School of Public Health, Ann Arbor, Michigan, United States

## Abstract

Recent studies have linked increased mortality to loneliness measured at a single point in time or over a short period of time, omitting the cumulative and dynamic nature of loneliness and its health effects. This study aimed to investigate long-term exposure to loneliness over an eight-year period in relation to subsequent all-cause mortality over 15 years. Data were from 9,032 individuals aged 50+ in the US Health and Retirement Study (HRS) from 1996-2019. Self-reported loneliness (yes; no) was measured biennially from 1996-2004. Cumulative loneliness was defined according to loneliness duration as never, intermittent (1-2 time points), and sustained (3+ time points). Mortality from 2004-2019 was identified from the HRS exit interviews and other sources (e.g., spouse). In the crude analysis, intermittent and sustained experience of loneliness was respectively associated with 137 (95%CI, 104-170) and 288 (95%CI, 233-343) excess deaths per 10,000 person-years, compared to those never feeling lonely. In the fully adjusted Cox proportional hazards model, a longer duration of loneliness was associated with additional 9% (HR=1.09, 95%CI, 1.02-1.17, intermittent vs. never) and 14% (HR=1.14, 95%CI, 1.03-1.27, sustained vs. never) mortality risk, indicating a dose-response relationship (P trend=0.009). Sustained loneliness may be a risk factor for mortality in mid-to-later life, and could be a potential intervention target to increase life expectancy.

